# Supplementary material for: A Pilot Study on the Effects of Transcranial Direct Current Stimulation on Brain Rhythms and Entropy during Self-Paced Finger Movement using the Epoc Helmet
Source: Front Hum Neurosci. 2017 Apr 25;11:201. doi: 10.3389/fnhum.2017.00201 (PMC5408787; doi:10.3389/fnhum.2017.00201)
Supplement: Supplementary file 1 [file Data_Sheet_1.zip › Complementary_results/Frequency_Bands_Rest-Referenced_average_PSD_windows/Gamma/Gamma_Band_Rest-Referenced_avg-win_P7.pdf]

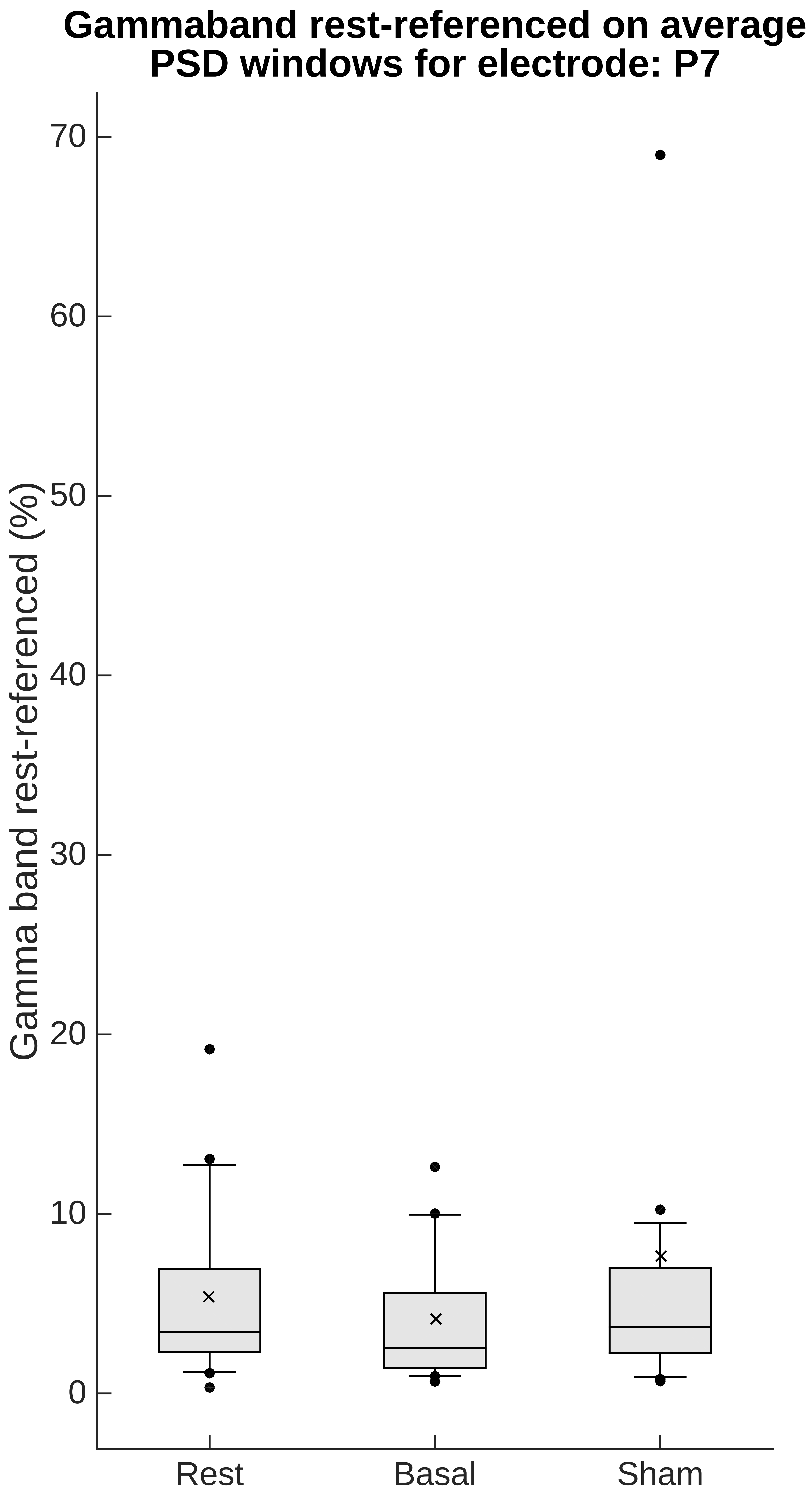

Gammaband rest-referenced on average
PSD windows for electrode: P7
Gamma band rest-referenced (%)
70
60
50
40
30
20
10
0
Rest
Basal
Sham
